# Intracellular C3 protects β-cells from IL-1β-driven cytotoxicity via interaction with Fyn-related kinase

**DOI:** 10.1073/pnas.2312621121

**Published:** 2024-02-12

**Authors:** Klaudia Kulak, Katarzyna Kuska, Lucie Colineau, Marina Mckay, Karolina Maziarz, Julia Slaby, Anna M. Blom, Ben C. King

**Affiliations:** ^a^Section of Medical Protein Chemistry, Department of Translational Medicine, Lund University, Malmö 214-28, Sweden

**Keywords:** diabetes, β-cell, IL-1β, FRK, C3

## Abstract

One of the hallmarks of type 1 but also type 2 diabetes is pancreatic islet inflammation, associated with altered pancreatic islet function and structure, if unresolved. IL-1β is a proinflammatory cytokine which detrimentally affects β-cell function. In the course of diabetes, complement components, including the central complement protein C3, are deregulated. Previously, we reported high C3 expression in human pancreatic islets, with upregulation after IL-1β treatment. In the current investigation, using primary human and rodent material and CRISPR/Cas9 gene-edited β-cells deficient in C3, or producing only cytosolic C3 from a noncanonical in-frame start codon, we report a protective effect of C3 against IL-1β-induced β-cell death, that is attributed to the cytosolic fraction of C3. Further investigation revealed that intracellular C3 alleviates IL-1β-induced β-cell death, by interaction with and inhibition of Fyn-related kinase (FRK), which is involved in the response of β-cells to cytokines. Furthermore, these data were supported by increased β-cell death in vivo in a β-cell-specific C3 knockout mouse. Our data indicate that a functional, cytoprotective association exists between FRK and cytosolic C3.

## Significance

Our research has uncovered a role of cytosolic C3 in safeguarding pancreatic β-cell function during inflammation, which is crucial for diabetes. We developed an animal model with C3 selectively absent in pancreatic β-cells to demonstrate its critical importance in preserving β-cell function and protecting against inflammation-induced β-cell death. This protective mechanism, mediated by cytosolic C3, counters the harmful effects of the proinflammatory cytokine IL-1β. These findings reveal an aspect of cytosolic C3 beyond its traditional association with the immune (complement) system, offering exciting possibilities for therapeutic interventions to preserve β-cell health and improve diabetes treatment.

Diabetes mellitus is characterized by a lack of or insufficient amount of insulin, resulting in loss of blood glucose homeostasis and chronic hyperglycemia. Type I diabetes (T1D) is caused by selective destruction of insulin-producing β-cells by autoimmune attack, while the much more prevalent type II diabetes (T2D) is associated with chronic low-level inflammation (1) and relative insulin deficiency. T2D can be divided into several phenotypic subtypes, which are defined partly by whether insulin deficiency or insulin resistance in target tissues is the primary cause of metabolic dysfunction (2).

Even though the causes and development of T1D and T2D are different, a common factor is the presence of components of innate immunity that are involved in progression of the disease (3). In particular, the activation of effector pathways involving interleukin-1β (IL-1β), which engages nuclear factor-κB (NF-κB) Jun N-terminal kinase (JNK) signaling, has been recognized as a critical driver of β-cell destruction (1, 4). Several studies aiming at the antagonizing of pathological IL-1β actions have shown benefits of the treatment in T2D that resulted in reduced levels of glycated hemoglobin (HbA1c) (5–7) and decreased risk of cardiovascular complications (8, 9). However, susceptibility to therapy is attenuated over time (9). In T1D, blocking of IL-1β activity was ineffective when used as a mono-immunotherapy, but combining it with adaptive immunity modulators remains an attractive approach (10). Nonetheless, still little is known about associated mechanisms accompanying IL-1β toxicity; thus, further investigations are important to enhance current approaches to preserve β-cell function.

Complement component 3 (C3) is a central molecule of the complement cascade that plays a significant role in innate immunity. Mature secreted C3 consists of a 75-kDa β-chain linked via a disulfide bond to the 115-kDa α-chain. Each of the three complement activation pathways (classical, lectin and alternative) lead to the generation of a C3 convertase, which cleaves C3 at the α-chain into the effector opsonin C3b, and the inflammatory modulator peptide C3a (11), providing powerful mechanisms for host defense against pathogens (12). However, the role of C3 is now known to extend beyond immune function, as it is also involved in intracellular communication systems and has an impact on cellular metabolism (13). Deregulation of the complement cascade has been described in various immune, inflammatory, and age-related conditions (14), but involvement of C3 in diabetes seems to be elusive. Expression of C3 is up-regulated in T1D pancreas (15) and T2D islets (16). Increased plasma levels of C3 have been associated with type 2 prediabetic (17, 18) and diabetic (19) states. A recent multitissue proteomics study, including islets, muscle, adipose tissue, liver, and blood, also found that an increase in inflammatory proteins in the islets, including complement proteins, gave one of the clearest indications of prediabetes, compared to healthy control donors (20). These findings suggest a role of islet complement in the early stages of T2D.

In both the human pancreas and in the INS-1 β-cell line, C3 was highly up-regulated and secreted under IL-1β (16) and IL-1β/TNF-α (21) treatments, suggesting a positive regulation by these cytokines in escalation of complement-mediated inflammation. Contrary to expectations, C3 has been reported to be a cytoprotective factor against β-cell death triggered by IL-1β and IFN-γ (21) and dysregulated autophagy (16), showing that as well as having a known proinflammatory role within innate immunity, C3 has additional beneficial roles in promoting β-cell survival and function. We previously identified the use of a noncanonical translational start site that can lead to cytosolic C3 expression in β-cells (16). Now we aimed to investigate the relevance of C3 and cytosolic C3 expression to β-cell survival during challenge by inflammatory cytokines.

## Results

### Intracellular C3 Is Up-Regulated under IL-1β Treatment in Human Pancreatic Islets and INS-1 β-Cells, and Promotes β-Cell Survival.

**Fig. 1.**
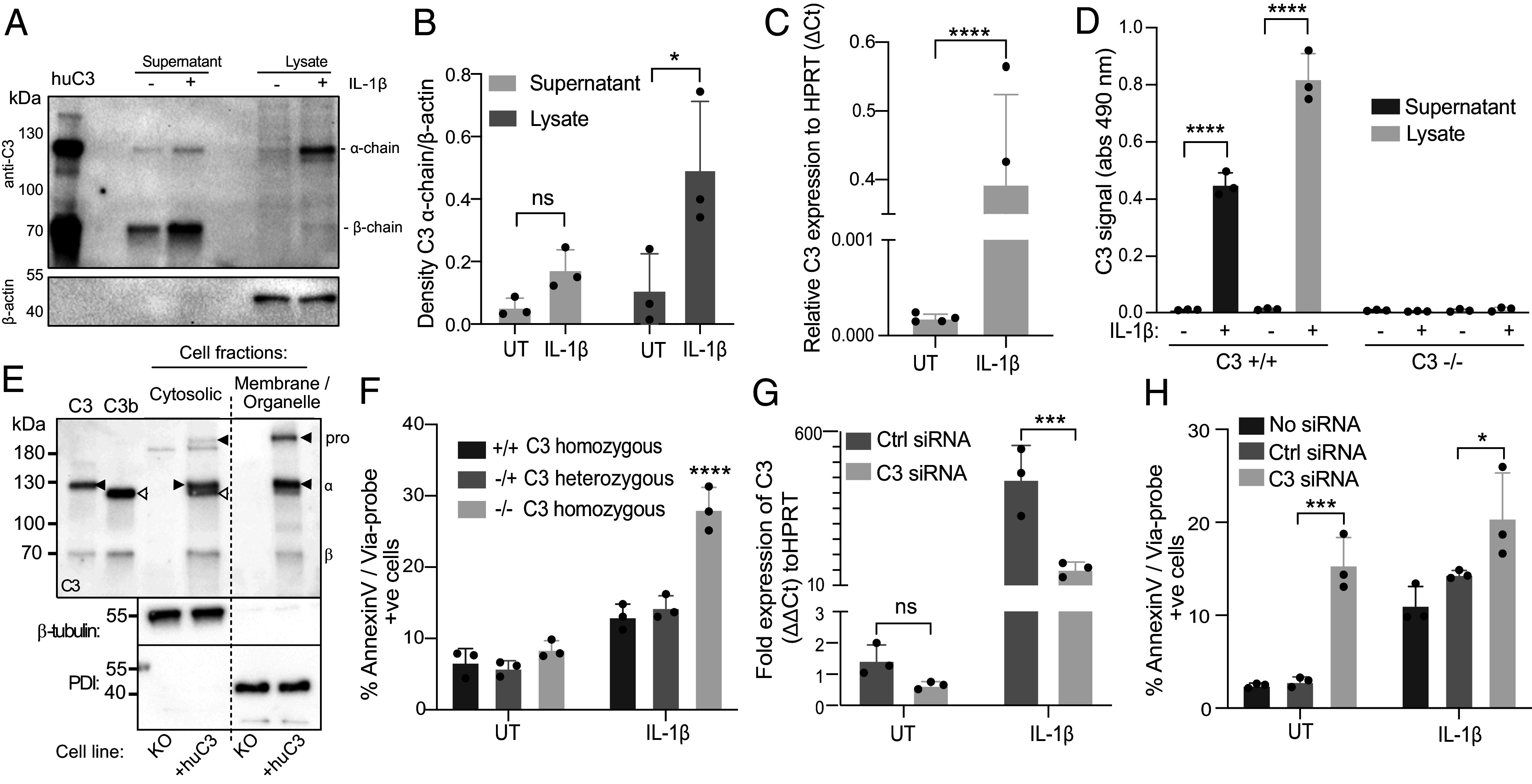
C3 is up-regulated after IL-1β treatment and is required for survival under IL-1β pressure. (*A*) Expression level of C3 protein in human pancreatic islets following overnight IL-1β treatment, representative blot of C3 protein in supernatants and lysates. (*B*) Quantification of C3 from (*A*) by densitometry. (*C*) mRNA expression level of C3 in INS-1 cells measured by qPCR showing great C3 upregulation after IL-1β treatment. (*D*) ELISA of rat C3 detection confirming upregulation of the C3 protein level in the supernatant and lysate of INS-1 WT clones. Lack of signals in C3 KO INS-1 clones (C3^−/−^) confirms specificity of C3 detection. (*E*) Blotting of cellular fractions from either C3-KO INS-1 cells or the same cells expressing human C3 (“+huC3”). The membrane/organelle and the cytosolic fractions from these cells were blotted for C3, as well as cytosolic marker, β-tubulin, or the ER marker, protein disulfide isomerase (PDI). Purified human C3 and C3b were loaded as size markers for C3 α- and β-chains. Pro-C3 and intact α-chain are marked with black arrowheads and the cleaved α′ of C3b with white arrowheads. Blot is representative of three independent repeats. (*F*) Viability of CRISPR/Cas9 INS-1 clones under IL-1β exposure. (*G*) Silencing efficiency of C3 siRNA treatment on C3 mRNA level, compared to ctrl siRNA. (*H*) C3 siRNA-treated cells suffer increased cell death under IL-1β treatment, confirming C3-specific gene targeting of CRISPR/Cas9 system and C3 deficiency-dependent reduction of cell viability. Bars display mean ± SD, with circles indicating individual repeats. (*C*) Two-tailed Student *t* test; (*B* and *D*–*G*) two-way ANOVA.

Previously, we reported high C3 expression in human pancreatic islets and found a significant increase of C3 secretion from human pancreatic islets after IL-1β treatment (16). It is now recognized that complement components also operate within the intercellular environment (22). To have an indication of what portion of C3 is up-regulated under IL-1β treatment, C3 levels were assayed in human islet culture supernatants and lysates. Upon IL-1β treatment, higher levels of C3 were found in islet supernatants, and significant upregulation of C3 protein was also observed in lysates (Fig. 1 *A* and *B*). IL-1β also up-regulated C3 gene expression levels in rat insulinoma cell line INS-1 832/13 (Fig. 1*C*), used as a model of β-cell function, and similarly to human pancreatic islets (16), IL-1β triggered INS-1 C3 secretion and enhanced generation of intracellular C3 protein found within lysates, which was undetectable in C3-KO INS-1 cells (Fig. 1*D*). To investigate the subcellular localization of C3 within β-cells, we produced INS-1 cells stably expressing human C3. On subcellular fractionation of these cells, C3 was found both within the membrane/organelle fraction, but also in the cytosolic fraction (Fig. 1*E*). Notably, within the cytosolic fraction, pro-C3 was largely processed to the mature α- and β-chains, but the α-chain was also cleaved to the same size as the α′ form found in C3b, providing evidence of cytosolic processing of C3 within β-cells.

IL-1β, if chronically overproduced, is involved in pancreatic inflammation and decline in β-cell function. To test the significance of C3 upregulation after IL-1β treatment, we investigated whether C3 affects the survival of INS-1 cells. Indeed, homozygous INS-1 clones deficient in C3 (C3^−/−^) exhibited increased apoptosis induced by IL-1β stimulation, while heterozygous loss of the C3 locus (C3^+/−^) produced a phenotype indistinguishable from homozygous wild-type (WT, C3^+/+^) clones (Fig. 1*F*). As an alternative approach to confirm higher sensitivity of INS-1 cells to IL-1β-induced cell death, and to exclude possible off-targets of the CRISPR/Cas9 system, we silenced C3 expression with siRNA. C3 knockdown (Fig. 1*G*) also resulted in a higher level of both basal and IL-1β-induced cell death (Fig. 1*H*). The enhanced cell death of C3 KO clones and C3-knockdown cells following IL-1β treatment therefore confirms a protective function of C3 against IL-1β-induced β-cell apoptosis.

### Complement Factor B is Expressed in Pancreatic β-cells and is Upregulated in Rodent Models of Diabetes.

**Fig. 2.**
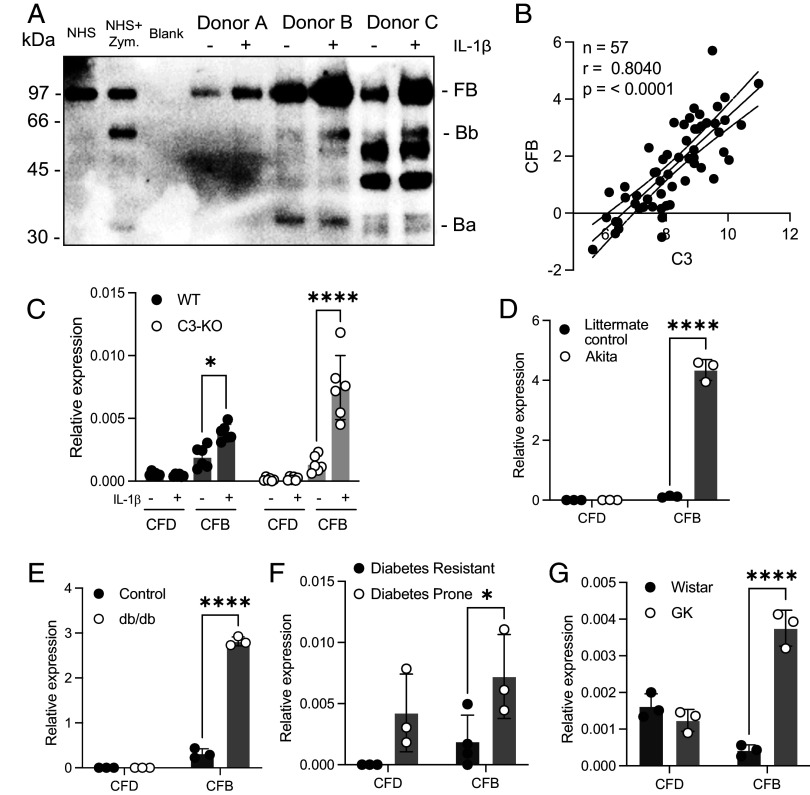
CFB and CFD expression is altered in human pancreatic islets, animal models of diabetes, and INS-1 C3-deficient clones. (*A*) WB for FB in supernatants from cultures of human pancreatic islets from three nondiabetic donors. NHS, normal human serum, with or without Zymosan (Zym) activation. (*B*) Correlation of RNA expression levels between C3 and CFB in 57 human islet preparations. (*C*) mRNA expression level of CFB and CFD in INS-1 WT and C3 KO clones, showing greater upregulation of CFB expression in C3 deficient clones measured by qPCR. (*D*–*G*) qPCR results of CFB and CFD expression in Akita (*D*) and db/db (*E*) mice, and diabetes prone BB (*F*) and GK (*G*) rats, showing upregulation of CFB in all tested diabetic models. Bars display mean ± SD with circles indicating three individual repeats from two individual clones (*C*) or number of animals (*D*–*G*). All statistical analyses are by two-way ANOVA.

Human pancreatic islets also express complement genes CFB and CFD (23), components of the alternative pathway C3 convertase, that is capable of cleaving C3 to release the C3a peptide from the α-chain. In previous experimental work showing an ameliorating effect of CFD and C3a on hyperglycemia and β-cell death, it was proposed that C3a production depends on FD and FB reaching the islets through the circulation (24). Given the appearance of cleaved C3 α-chain in INS-1 cytosolic fractions (Fig. 1*E*), we investigated the potential for autocrine alternative complement pathway activation in pancreatic islets. We tested expression of factor B and factor D in islets and INS-1 cells under an inflammatory milieu representing the diabetic state. Factor B secretion into the supernatant of human pancreatic islets increased following IL-1β treatment, and the detectable activated Bb fragments of Factor B in two of three tested donors indicate activation of the alternative pathway of complement (Fig. 2*A*). Human islet C3 expression is not only up-regulated by IL-1β treatment but also correlates strongly with islet IL-1β expression, donor BMI and diabetic status (16). Likewise, in human islet expression data, CFB correlated strongly with C3 (Fig. 2*B*). Indeed, using the Islet Geneview online web resource referencing RNA expression data from 188 human islet donors (25), CFB ranked higher than any other gene as correlating most significantly with C3 expression (*SI Appendix*, Table S1). In RNA expression data from fresh human islets, C3 and CFB, but not CFD, correlated most strongly with islet cytokine expression levels, while CFD expression correlated only with BMI, and IL-6 (*SI Appendix*, Fig. S1).

mRNA expression levels of CFB, but not CFD, were also up-regulated by IL-1β treatment in INS-1 cells (Fig. 2*C*). Although CFB and CFD levels correlate highly in human islets, CFB upregulation by IL-1β is not C3 dependent, as shown by its upregulation in C3 KO INS-1 cells. CFB mRNA was also clearly up-regulated in isolated pancreatic islets of all tested animal models of diabetes: Akita and db/db mouse models for phenotypes associated with T1D and T2D, respectively, and BB and GK rats, which correspond to T1D and T2D models (Fig. 2 *D*–*G*). CFD expression was very low or not detected in most mouse samples, or had no significant difference between healthy and diabetic rats. Our data therefore demonstrate that both islet C3 and CFB are up-regulated in diabetic islets, allowing the potential for alternative pathway activation independently from factors produced outside of the islets, although low levels of CFD expression in mice and humans may be a limiting factor.

### IL-1β-Mediated Increased Death of C3 KO Clones Is Not Rescued by Exogenous C3 or C3aR Signaling.

**Fig. 3.**
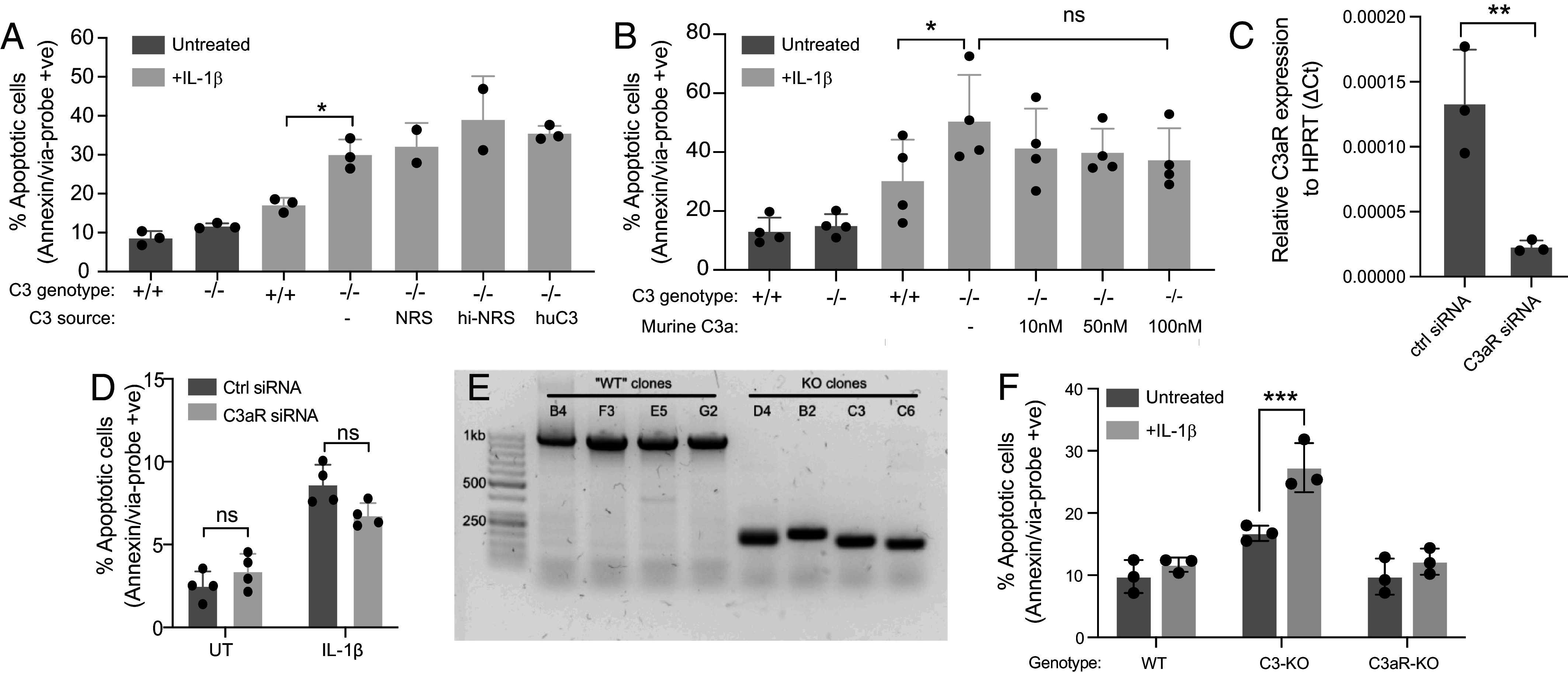
Exogenous C3 or C3a does not rescue IL-1β-induced apoptosis in C3 KO INS-1 cells. (*A*) Viability of INS-1 WT and C3 KO clones after IL-1β treatment showing that exogenous addition of different sources of C3 either from NRS/HI-NRS or purified C3 has no effect on survival of C3-deficient clones. (*B*) Viability of INS-1 clones, showing no significant effect C3a addition after IL-1β treatment. (*C*) qPCR of C3aR KD efficiency in INS-1 cells. (*D*) Viability of INS-1 cells after C3aR KD. (*E*) Agarose gel of PCR products from genomic DNA using primers for the C3aR locus, demonstrating C3aR knockout. (*F*) Viability of INS-1 clones treated with IL-1β, as assessed by flow cytometry, showing no effect of C3aR KO. Bars display mean ± SD with circles indicating individual repeats. Statistics in *A* and *B*, one-way ANOVA; in *D* and *F*, two-way ANOVA; in *C*, two-tailed *t* test.

Since alternative pathway components are up-regulated in diabetic human and rodent islets, which could lead to C3 cleavage and release of the C3a peptide from the C3 α-chain, we tested potential roles of C3a signaling on survival of C3 KO clones, by exogenous addition of serum or C3/C3a. Treatment of C3 KO clones with normal rat serum (NRS), where C3 is abundantly present (26), or heat-inactivated (HI) NRS, or purified human C3, did not rescue the decreased viability of C3 KO clones in the presence of IL-1β (Fig. 3*A*). Differences in apoptosis observed after addition of a titration of recombinant murine C3a were also not statistically significant (Fig. 3*B*). siRNA-mediated knockdown of the C3a receptor (C3aR) in INS-1 cells, resulted in significant silencing of C3aR gene expression in unstimulated WT INS-1 832/13 cells (Fig. 3*C*), but had no effect on their viability when challenged with IL-1β (Fig. 3*D*), suggesting that the decreased viability in IL-1β-treated C3 KO cells is not due to reduced C3aR signaling. To further support this hypothesis, we used CRISPR/Cas9 to create C3aR-KO INS-1 clones, using a strategy to remove roughly 800 base pairs from the coding region of the genomic C3aR locus. PCR using flanking primers confirmed this genomic deletion in multiple clones (Fig. 3*E*), but C3aR-KO INS-1 cells again showed no increase in apoptosis in comparison to WT clones, when exposed to IL-1β (Fig. 3*F*), confirming that the prosurvival effect of cell-intrinsic C3 is independent of C3a signaling via C3aR.

### IL-1β-Mediated Apoptosis Induction Is Inhibited by Cytosolic C3.

**Fig. 4.**
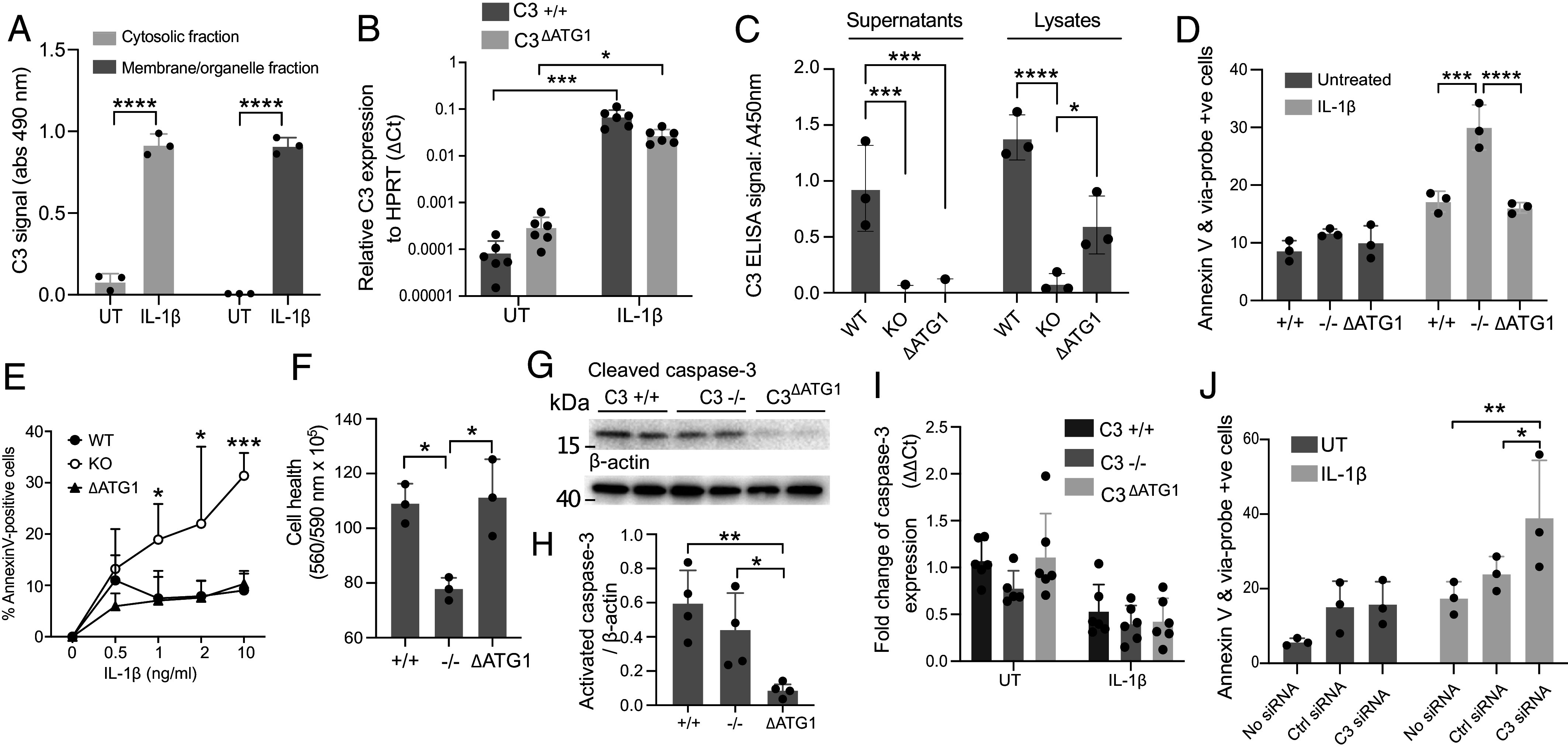
Expression of cytosolic C3 is protective against IL-1β-induced apoptosis in INS-1 cells. (*A*) ELISA for C3 in cellular fractions of INS-1 cells treated with or without IL-1β, demonstrating upregulation in both organelle and cytosolic fractions. (*B*) mRNA expression level of C3 in WT and ΔATG1 INS-1 clones measured by qPCR. (*C*) ELISA for C3 in supernatants and cell lysates of INS-1 clones after IL-1β exposure. (*D*) Viability of INS-1 clones upon overnight incubation with 1 ng/mL IL-1β, showing complete ΔATG1-C3-mediated rescue of reduced cell survival of C3-deficient clones. (*E*) Apoptosis induced by overnight incubation of INS-1 clones with a titration of IL-1β, as measured by % of Annexin-V binding cells above background. (*F*) Alamar Blue viability assay confirms reduced health of C3 KO and improved survival of ΔATG1-C3 expressing cells after IL-1β addition. (*G*) Representative blot of activated caspase-3 detection in multiple IL-1β-treated gene-edited clones, showing low level of activated caspase-3 in ΔATG1 clones. (*H*) Quantification of densitometry from four repeats of western blots for active caspase 3. (*I*) mRNA expression level of caspase-3 in multiple INS-1 clones, measured by qPCR. (*J*) ΔATG1-C3 expressing INS-1 clones suffer increased cell death after C3 silencing, supporting cytosolic C3 involvement. Bars display mean ± SD with points indicating individual repeats, except of *D*, *F*, and *H*, where points are representative of three individual repeats from two INS-1 clones. (*F* and *H*) one-way ANOVA; (*A*, *B*, *D*, *E*, *I*, and *J*) two-way ANOVA.

We previously identified intracellular cytosolic C3 as a regulator of cytoprotective autophagy in β-cells (16). IL-1β treatment causes increased secretion but also intracellular content of C3 (Fig. 1*C*). Increased levels of intracellular C3 protein might represent a membrane-bound fraction destined for secretion, or a cytosolic pool that might indicate participation in regulating intracellular events (27, 28). Subfractionation of INS-1 cells into membrane and cytosolic fractions, followed by ELISA, revealed IL-1β-mediated upregulation of C3 protein in both analyzed fractions (Fig. 4*A*).

To investigate whether the cytosolic pool of C3 is also involved in the β-cell response to IL-1β treatment, we used CRISPR/Cas9 to introduce a single nucleotide indel mutation within the genomic coding sequence of the C3 signal peptide, immediately downstream of the canonical ATG start codon, leading to a frame-shift mutation and premature stop codon (*SI Appendix*, Fig. S2). Although this prevents functional expression and secretion of canonical C3, in other cell lines this has previously been proven to still allow expression of a nonsecreted, cytosolic C3 isoform from an in-frame start codon downstream of the signal peptide (16, 29). IL-1β still induced upregulation of C3 mRNA in INS-1 clones with a nonfunctional canonical translational start site (ΔATG1 cells) (Fig. 4*B*), and while ΔATG1 cells no longer secreted C3, IL-1β treatment still caused its intracellular accumulation (Fig. 4*C*). C3 expression in ΔATG1 cells also rescued IL-1β-induced apoptosis to levels comparable with WT clones, as measured by AnnexinV binding in flow cytometry (Fig. 4*D*). This was true over a range of IL-1β concentrations, with WT and ΔATG1 INS-1 clones showing the same susceptibility to IL-1β-induced apoptosis, while C3-KO clones underwent increasing amounts of apoptosis in a dose-responsive fashion (Fig. 4*E*). The rescuing of IL-1β-induced apoptosis by the cytosolic expression of C3 in ΔATG1 cells was also verified by colorimetric Alamar blue viability assay, which measures the redox capacity of live cells (Fig. 4*F*). Unexpectedly, significantly lower levels of activated caspase-3 were found in ΔATG1 clones compared to WT and C3 KO clones (Fig. 4*G*, quantified in 4H) while levels of caspase-3 mRNA expression were not changed (Fig. 4*I*), ruling out that observed changes are a result of different amounts of pro-caspase-3. Finally, to verify that cytosolic C3 expressed in the IL-1β-treated ΔATG1 gene-edited clones had a prosurvival function, ΔATG1 cells were treated with C3-targeting siRNA. Knock-down of C3 mRNA also enhanced apoptosis in ΔATG1 cells exposed to IL-1β (Fig. 4*J*), confirming that intracellular cytosolic C3 mediates a protective response against IL-1β.

### C3 Interacts with Proapoptotic Fyn-Related Kinase (FRK).

Previously, we demonstrated a protective role of intracellular C3 against dysregulated autophagy in β-cells, via interaction with autophagy-related protein 16-1 (ATG16L1) (16). Since IL-1β did not induce autophagy within 24 h of incubation, as no increased level of autophagy marker LC3-II was observed either with or without the lysosomal inhibitor chloroquine (Chq, *SI Appendix*, Fig. S3 *A* and *B*), we hypothesized that the mechanism by which IL-1β contribute to higher cell death of C3^−/−^ clones is not autophagy-related. C3 signaling may also be involved in regulation of ATG16L1 expression, as reported recently in significantly changed genes across C3a-treated INS-1 cells (30). However, C3 knockout did not affect ATG16L1 expression levels in cells used in our experiments (*SI Appendix*, Fig. S3*C*).

**Fig. 5.**
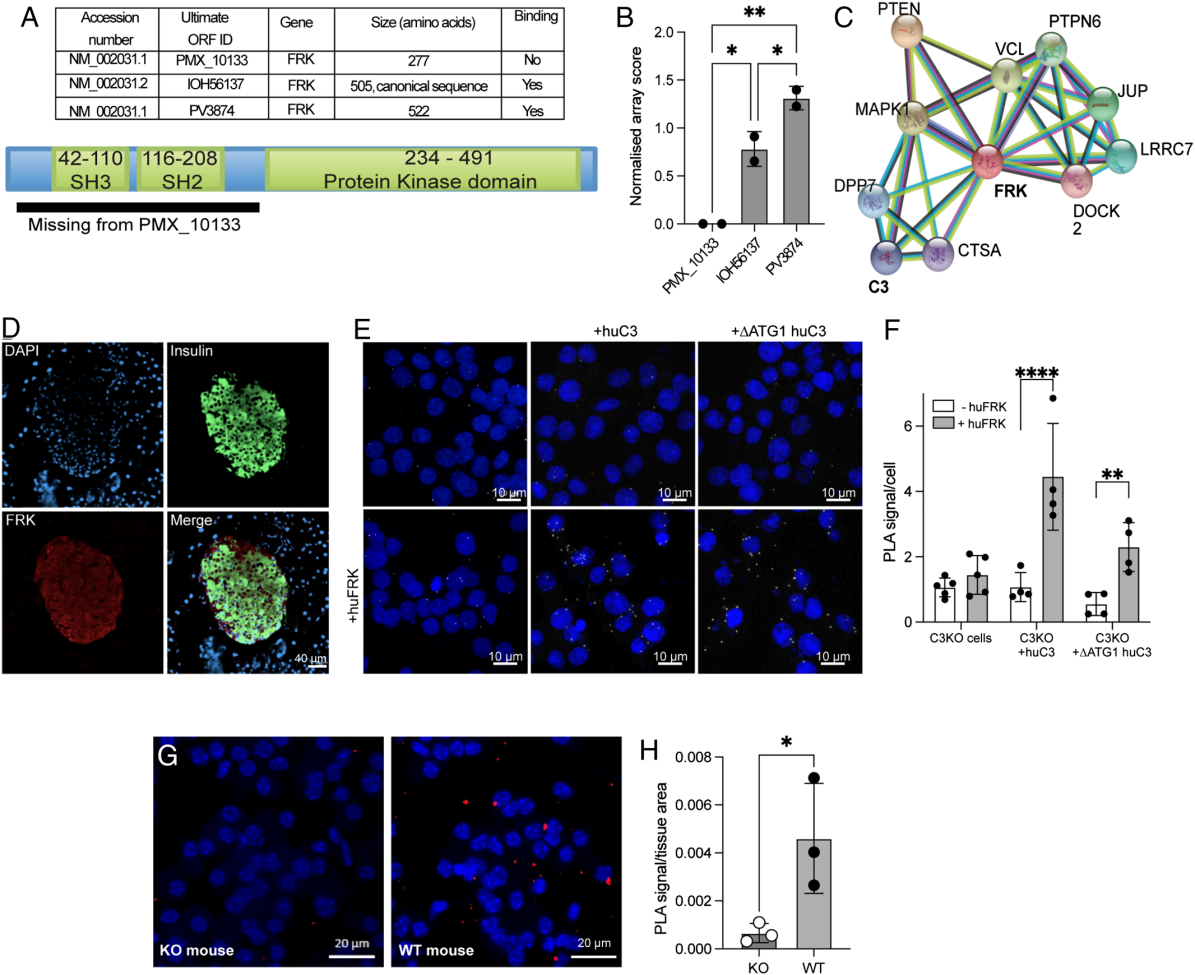
C3 and FRK interact within islet cells. (*A*) Isoforms of FRK bound by protein microarray: Table showing accession numbers of FRK spots on the protein microarray and their interaction with serum-purified human C3, with a map of FRK below. (*B*) Normalized protein array scores for individual FRK isoforms from two microarray repeats. (*C*) String.org protein–protein interaction network for FRK. Black line—coexpression of putative C3 and FRK homologous in *Drosophila melanogaster, Mus musculus, Pseudomonas aeruginosa,* and *Rattus norvegicus*; blue line—association/interaction from curated database; green line—text mining interaction source. (*D*) Staining of mouse pancreas sections with anti-FRK antibody, confirming islet-specific expression patterns. (*E*) PLA results from C3-KO INS-1 cells transfected with either WT huC3 plasmid or ΔATG1-human C3 plasmid, plus (below) or minus (above) human FRK plasmid cotransfection. Colocalization was found between both total C3 and ΔATG1 (cytosolic) C3. (*F*) Quantification of C3/FRK PLA results, from three independent repeats. Blue: DAPI staining of nuclei, white: colocalization puncta between C3 and FRK. (*G*) Representative image of PLA for C3 and FRK from isolated mouse islets. Red: colocalization puncta. (*H*) Quantification of PLA signal between C3 and FRK in WT or C3-KO mouse islets (three mice per group). Bars display mean ± SD. (*B*) one-way ANOVA; (*F*) two-way ANOVA; (*H*) unpaired *t* test. (Scale bar in *D*, 20 µm.)

Our previous ProtoArray protein interaction microarrays revealed binding of C3 to two isoforms of FRK (16), but not to a third isoform that lacks the N-terminal SH3/SH2 domains (Fig. 5 *A* and *B*), implicating this as the site of C3 binding. Searching the STRING database for FRK predicted functional partners also revealed C3 (Fig. 5*C*), supporting the possibility of a C3-FRK interaction. FRK (also known as GTK/RAK/BSK/IYK) has been implicated in apoptosis induction in islet cells by proinflammatory cytokines (31–33), and staining for FRK in mouse pancreatic sections revealed an islet-specific expression pattern (Fig. 5*D*). When human FRK was introduced into INS-1 cells over-expressing human C3, proximity ligation assay (PLA) demonstrated a close (<50 nm) colocalization of these proteins (*SI Appendix*, Fig. S4). This was confirmed when cotransfecting INS-1 cells with human FRK plasmid, and plasmids containing either WT (canonical) or ΔATG1 human C3 (Fig. 5 *E* and *F*), showing that cytosolic C3 and FRK associate together within INS-1 cells. PLA assay of primary isolated mouse pancreatic islets also demonstrated colocalization between C3 and FRK, with no PLA signal from C3 KO mouse islets as negative controls (Fig. 5 *G* and *H*). Together with the known established role of FRK in mediating islet cell cytokine-induced cytotoxicity, these data suggested a hypothesis that a C3/FRK interaction mediates the protective effect of C3 within β-cells.

### C3 Regulates FRK Levels and IL1R Downstream Signaling.

**Fig. 6.**
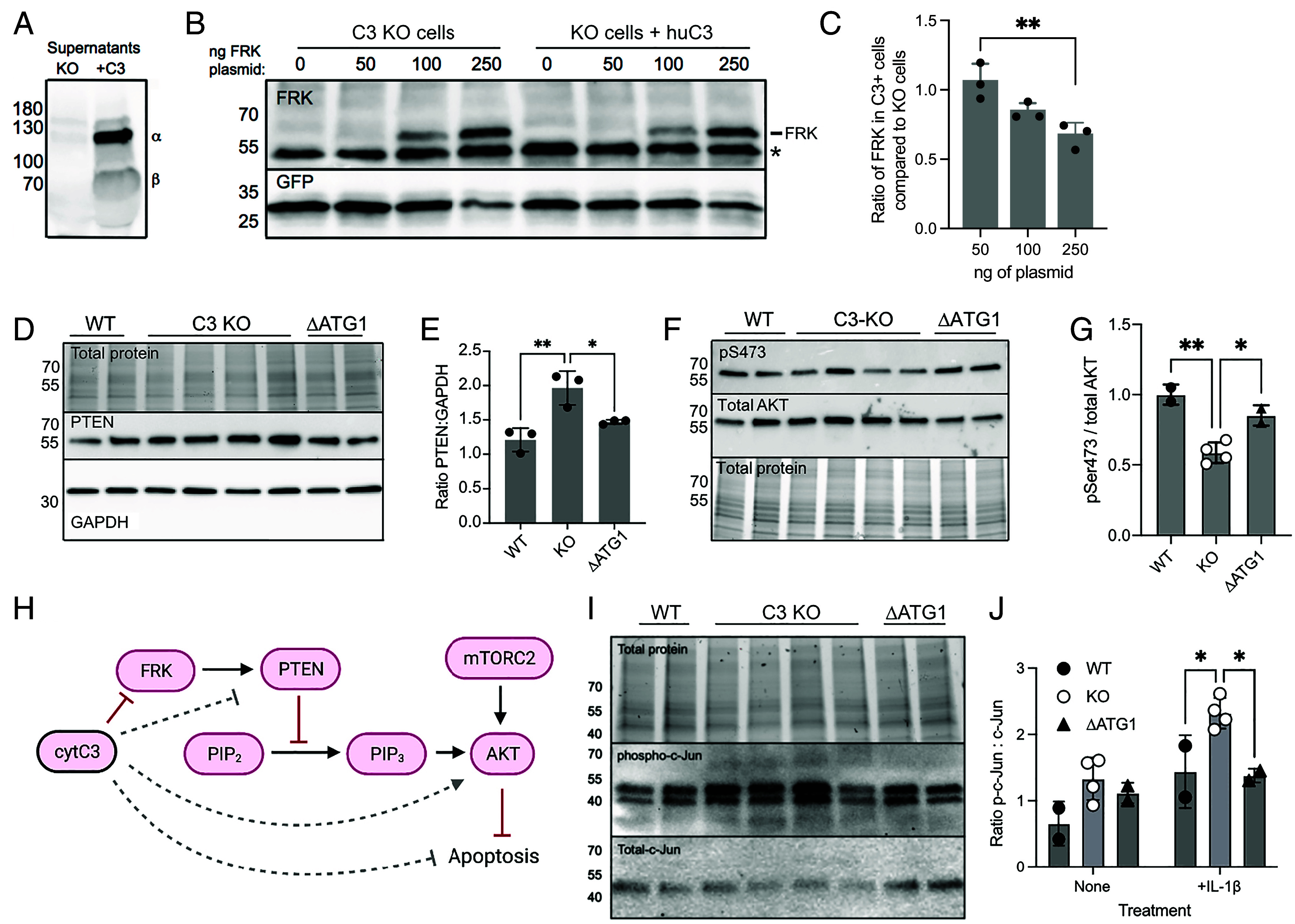
C3 expression alters FRK expression and subsequent downstream signaling. (*A*) Western blot for C3 in supernatants of transfected INS-1 clones. (*B*) Western blot for cotransfected FRK and EGFP in INS-1 and INS-1-huC3 cells, with titration of the FRK plasmid. A nonspecific band also seen in untransfected cells is marked with an asterisk (*). (*C*) Ratio of the FRK signal in C3 KO compared to C3-expressing cells, after normalization to EGFP transfection control, from (*B*). (*D*) Western blot for total PTEN in lysates from multiple WT, C3-KO, or ΔATG1 INS-1 clones. (*E*) Quantification of PTEN amounts normalized to total loaded protein, from three independent repeats. (*F*) Western blot for phospho-AKT and total AKT levels in lysates of multiple WT, C3-KO and ΔATG1 INS-1 clones exposed to 1 ng/mL IL-1β for 30 min. (*G*) Quantification of AKT phosphorylation as shown in (*F*), from three independent repeats. (*H*) Scheme of suggested role of cytosolic C3 in regulation of FRK and downstream signaling in β-cells. Direct interactions or inhibitions are shown by unbroken lines, indirect effects are indicated by dashed lines. CytC3, cytosolic C3. (*I*) Western blotting for c-JUN phosphorylation in multiple INS-1 clones exposed to 1 ng/mL IL-1β for 30 min. (*J*) Quantification of phospho-cJUN in individual clones before and after IL-1β stimulation, from three independent repeats. Statistics in *C*, *E*, and *G*, one-way ANOVA, and *J*, two-way ANOVA, with Bonferroni posttests.

To investigate whether C3 expression influences functions of FRK, we first tested FRK expression levels. A plasmid containing the cDNA for human FRK was successfully transfected into INS-1 cells that were either C3-KO or stably expressing human C3, as confirmed by western blot (Fig. 6*A*). To investigate potential C3-mediated specific suppression of FRK, lysates were then blotted for both FRK and cotransfected EGFP (Fig. 6*B*), and the ratios were assessed by densitometry. Cells expressing human C3 had a 30.2 ± 9.3 % reduction of FRK western blot signal strength, compared to the C3 KO parental clone (Fig. 6*C*), suggesting that C3 may directly attenuate FRK levels.

FRK interacts with the phosphatase and tensin homolog (PTEN) via its N-terminal SH3 domain, and phosphorylates PTEN at Y336, thereby preventing its degradation by the ubiquitinylation pathway (34). Loss of C3 and an increase in FRK function would therefore be expected to lead to an increase in PTEN protein levels. Lysates from WT, C3-KO, and ΔATG1 INS-1 clones were therefore blotted for total PTEN levels. Consistent with a role for C3 in suppressing FRK, C3-KO INS-1 clones contained higher levels of total PTEN (Fig. 6 *D* and *E*). PTEN itself negatively regulates activation of the prosurvival factor AKT, via dephosphorylation of the second messenger PIP3, which recruits AKT to the cell membrane where it is phosphorylated at Serine473 by mTOR complex 2 (mTORC2) (35). Consistent with this function, AKT in IL-1β-stimulated C3-KO INS-1 clones also had lower rates of phospho-Ser473 (Fig. 6 *F* and *G*). In all cases, ΔATG1 cells had a reversed phenotype of KO cells, supporting a role for cytosolic C3 in regulation of FRK, that impacts on subsequent downstream signaling (Fig. 6*H*).

As well as stimulating the PI3-kinase pathway, leading to PIP3 formation and AKT signaling, IL1R stimulation also triggers the p38/MAPK pathway. To investigate the downstream outcome of IL1R signaling on this pathway in C3 KO cells, we tested lysates of IL-1β-exposed INS-1 clones for phosphorylation of cJUN, that, when activated, enters the nucleus to engage a proapoptotic gene expression program resulting in caspase activation (36). After incubation with IL-1β, C3 KO clones experienced increased levels of cJun phosphorylation compared to either WT or ΔATG1 clones (Fig. 6 *I* and *J*), again implicating cytosolic C3 as a protective, anti-apoptotic pool of C3 that can regulate proapoptotic signaling. Thus, C3 appears to affect the activity of FRK, translating into downstream effects of the PI3K pathway, as well as affecting outcomes of p37/MAPK signaling as seen by effects on cJUN phosphorylation.

### β-Cell-Specific Loss of C3 Accelerates Diabetes Development in STZ-Treated Mice.

**Fig. 7.**
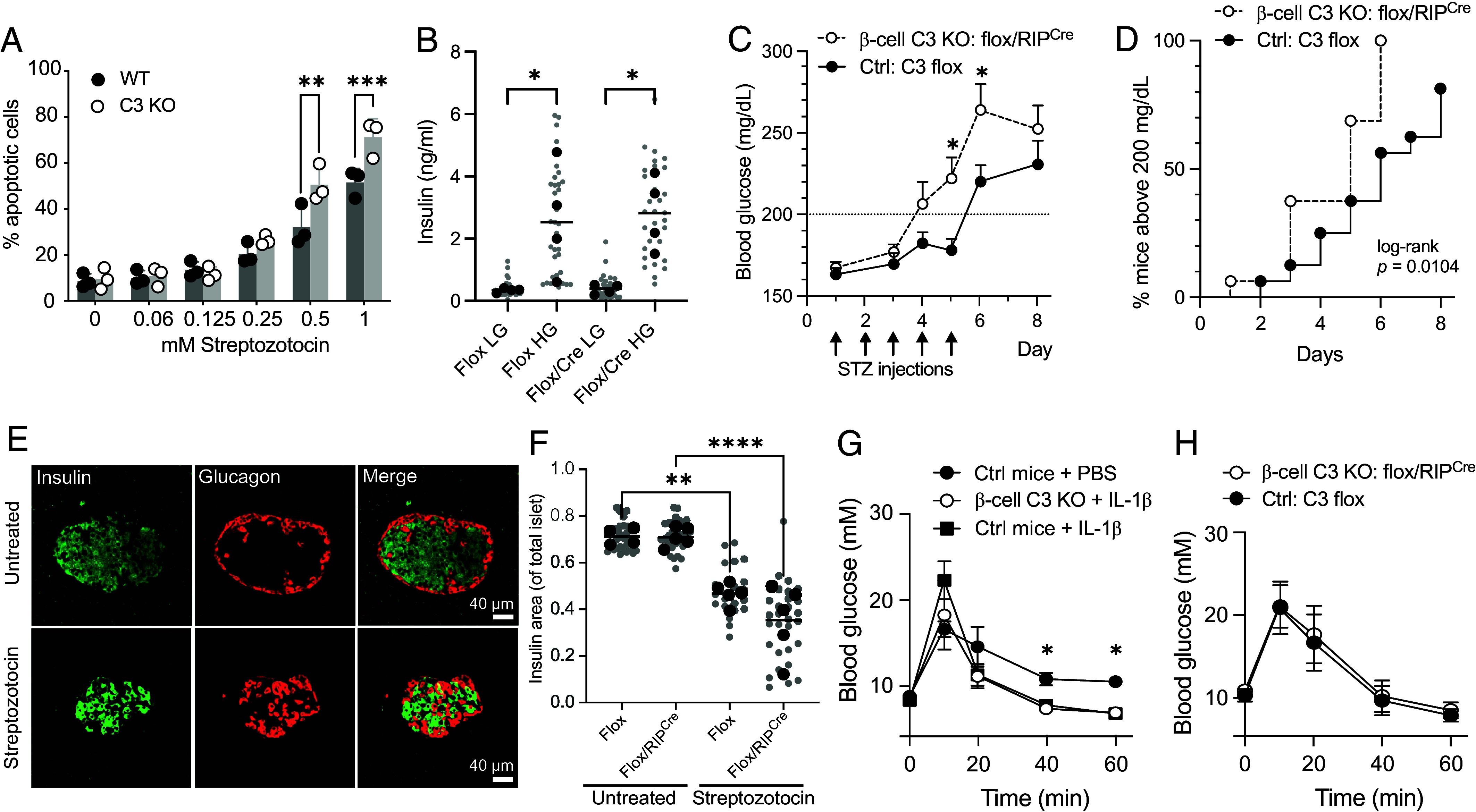
β-cell-specific C3 knockout affects detrimental but not beneficial effects of IL-1β. (*A*) Apoptosis measured by annexin-V binding in WT or C3-KO INS-1 cells after exposure to STZ. (*B*) Glucose stimulated insulin secretion from isolated islets from floxed or flox/RipCre mice. Isolated islets were incubated in low (LG) or high glucose (HG) conditions for 1 h, and secreted insulin measured by ELISA. Islets were isolated from four mice per group. Large black symbols are averages per mouse, individual islet secretions are shown as small symbols in gray. (*C*) Average blood glucose levels in β-cell-specific C3-KO mice or littermate controls, after receiving multiple low-dose STZ. *n* = 16 mice per group. (*D*) Development of diabetes (blood glucose > 200 mg/dL) in mice from (*C*). (*E*) Representative examples of islets from pancreatic sections stained for insulin and glucagon, before and after multiple low-dose streptozotocin treatment, demonstrating loss of insulin-positive β-cells. (*F*) Ratio of insulin as total proportion of islet, in islets analyzed from Flox and flox/RIP-Cre mice before or after streptozotocin treatment. Large icons are averages per mouse; small icons are individual islet values. (*G*) Blood glucose measurements in mice receiving IP glucose injection, with or without preinjection of PBS or IL-1β. Results from two repeats in female mice, with *n* = 3 per group per repeat. (*H*) Blood glucose measurements in male mice receiving IL-1β injection followed by IP glucose. *n* = 4 (flox controls) or 5 (flox/Rip^Cre^) per group. Results show mean ± SEM except (*A*, *B*, and *F*), mean ± SD. Statistics show two-way ANOVA, except (*D*), log-rank test.

We next tested the induction of β-cell death in vivo. β-cell-specific overexpression of FRK renders mice more susceptible to diabetes induction after treatment with streptozotocin (STZ) (37), a glucose homologue that is taken up by the GLUT2 transporter on β-cells and leads to DNA alkylation and apoptosis induction. As FRK is involved in STZ-induced β-cell apoptosis, we therefore hypothesized that if C3 inhibits FRK function, loss of C3 would also increase STZ susceptibility. Incubation of C3-KO INS-1 cells with STZ in vitro led to enhanced levels of apoptosis as compared to WT C3-expressing cells (Fig. 7*A*). We then generated β-cell-specific C3 KO mice by crossing floxed C3 mice (38) with mice expressing the Cre recombinase under control of the rat insulin promoter (RIP-Cre), both on the C57Bl/6 N background. The resultant mice lack β-cell C3 expression but have identical levels of serum C3 as floxed littermates. We found no difference in the basal function of isolated islets from these mice under healthy conditions, which secreted equal amounts of insulin when stimulated with high glucose (Fig. 7*B*). In order to challenge β-cells in vivo, these mice were then subjected to a multiple low-dose (MLD) streptozotocin model of diabetes induction, which produced a significantly more rapid blood glucose elevation in flox/RIP-Cre β-cell-specific C3 knockout mice, as compared to floxed littermate controls (Fig. 7*C*), indicative of enhanced β-cell death and loss of insulin production. This translated to a more rapid onset of diabetes in these same mice (Fig. 7*D*). Loss of β-cell-intrinsic C3 expression therefore recapitulated the same phenotype as β-cell-specific FRK overexpression (37), rendering β-cells more vulnerable to apoptosis, despite the unchanged levels of extracellular serum C3. Histological analysis of pancreases from these mice showed no difference in overall islet size or insulin-positive volumes in untreated mice between the two genotypes, whereas STZ treatment resulted in reduction in overall islet size, with loss of insulin-positive β-cells (Fig. 7*E*), and a significant reduction in insulin-positive cells as proportion of entire islets (Fig. 7*F*). There was a trend toward higher insulin loss in STZ-treated β-cell-specific C3-KO mice, consistent with increased β-cell susceptibility to apoptosis.

Although chronic elevated IL-1β levels contribute to islet inflammation and β-cell apoptosis, and islet expression of IL-1β correlates with diabetes development (39), short-term production of small amounts of IL-1β by peritoneal macrophages also contributes to enhanced postprandial insulin secretion (40). To assess whether intrinsic expression of β-cell C3 altered this augmentation of insulin secretion in response to low-level IL-1β in vivo, mice were injected IP with 1 ng/g IL-1β 20 min before glucose challenge. As expected, preinjection with low levels of IL-1β resulted in significantly enhanced clearance of blood glucose in female mice (Fig. 7*G*), while there was no difference in this enhanced clearance when comparing flox/RIP-Cre (β-cell-specific KO) and control floxed mice. A second experiment in male mice also showed no difference between genotypes (Fig. 7*H*). β-cell intrinsic C3 therefore appears to regulate the negative, proapoptotic effect of IL-1β exposure, without affecting the beneficial ability for low levels of IL-1β to augment insulin secretion.

## Discussion

Pancreatic islet inflammation is a feature of both T1D and T2D (1), and proinflammatory cytokines adversely affect β-cell function and viability, contributing to insufficient insulin secretion and loss of blood glucose homeostasis. The identification of factors that maintain β-cell function, and their mechanisms of action, is therefore important in identifying potential strategies for supporting insulin secretion in diabetic individuals. We previously showed that as well as being secreted, C3 can be found in the cytosolic fraction of cells (16, 27) where it can interact with cytosolic proteins such as ATG16L1, and we have identified two pathways, retrotranslocation as well as translation initiation from a noncanonical translational start site downstream of the signal peptide, by which C3 could enter the cytosol (27, 28). By CRISPR/Cas9 editing of cellular genomes to introduce an indel directly after the canonical start codon, we have produced cell lines that express C3 lacking a signal peptide, therefore preventing C3 secretion, but allowing its expression in the cytosol, thereby creating a tool by which to study its cytosolic-specific functions. Here we have found not only that loss of C3 expression renders β-cells more vulnerable to apoptosis induction by IL-1β, but identified that this is due to a function of the cytosolic pool of C3, via interaction with FRK.

We previously found that C3 is highly up-regulated in isolated pancreatic islets from human diabetic donors, as well as in many rodent models of diabetes (16). We now show that CFB is also significantly up-regulated in isolated islets of several rodent models of diabetes, is secreted from isolated human islets, where it becomes cleaved to Bb, a sign of activation. The alternative pathway of complement is canonically initiated by slow conversion of C3 to C3_H2O_, via hydrolysis of the internal thioester group of C3. This causes a conformational change, allowing binding of factor B, which is then cleaved by factor D to Bb. The complex of C3_H2O_Bb forms a C3 convertase which can cleave further copies of C3 into C3b, releasing C3a, and allowing further convertase formation by the binding of factor B to C3b. We observed only very low levels of factor D expression in isolated islets and no detectable expression in INS-1 cells. It is therefore possible that an additional factor is responsible for conversion of factor B to Bb in human islet supernatants. C3 itself appears to be converted to C3b within INS-1 cytosolic fractions, and we are currently investigating which factors may be responsible for this. Regardless of the exact initiation mechanism, C3 convertase action cleaves C3 to release the C3a peptide, which has been shown to augment insulin secretion (24). However, we found no evidence that C3a was responsible for increased survival in β-cells, as tested by addition of recombinant C3a, or removal of the C3a receptor.

In our previous study, an investigation into C3-protein interactions by protein microarray revealed FRK as one of the top binding partners (16). FRK is a tyrosine kinase expressed in pancreatic islets (32) and implicated in survival and growth of β-cells, having previously been shown to be involved in cytokine- and STZ-induced β-cell apoptosis (31), with FRK knockout or inhibition making cells more resistant (31), while over-expression renders cells more vulnerable to apoptosis induction (33, 37). Our protein microarrays suggest the N-terminal SH3 domain of FRK as the probable C3 interaction site. This is also the site by which FRK interacts with PTEN (34). β-cell-specific deletion of PTEN phenocopies knockout of FRK, leading to STZ resistance in vivo (41). We therefore hypothesize that the C3 interaction with FRK, confirmed here in clonal cells and primary mouse islets by PLA, inhibits the FRK/PTEN interaction, leading to reduction of FRK and PTEN at the protein level (Fig. 6 *C* and *E*), that is reversed in C3 KO cells. Increases in PTEN cause reduction in AKT signaling and subsequent reduced β-cell survival. Activation of AKT is prosurvival and has been described to inhibit apoptosis at the postmitochondrial level, but upstream of caspase 9 and 3 activation (42), and so the decrease in AKT phosphorylation in C3-KO cells is consistent with the increase in apoptosis seen in C3-KO cells after IL-1β exposure.

The phenotype of β-cell-specific C3 knockout mice when exposed to the STZ model of diabetes induction was consistent with this hypothesis and in line with demonstrated phenotypes of FRK and PTEN deletion. It should be noted that whole body knockout of C3 conveys a resistance to multiple low-dose STZ induction of diabetes development after 6 to 7 d (43). This was however identified as due to the role of C3 in the adaptive immune system, as multiple low dose-STZ induces a form of diabetes that is transferable via adoptive transfer of T cells from a multiple low dose-STZ-treated to an untreated mouse. Therefore, while C3 intrinsically expressed by β-cells is protective, C3 expressed by immune cells is involved in development of an adaptive T cell response to autoantigens released due to β-cell destruction (43). As our model contains a β-cell-specific C3 deletion, with identical levels of serum C3 in specific knockouts and littermate controls, we can attribute the STZ sensitivity in our mice to the loss of β-cell-intrinsic C3 expression.

Cytosolic expression of C3 in ΔATG1 cells resulted in a complete rescue of the reduced cell survival of C3-KO clones when exposed to IL-1β. Previous reports have been made of C3a-mediated preservation of β-cell function in diabetic mice (30), and protective roles of C3a against IL-1β/IFNγ-induced apoptosis in INS-1E cells (21). In our hands, extracellular addition of C3a did not make statistically significant changes in cell survival, although there was a nonsignificant trend toward improved survival of C3^−/−^ clones (Fig. 3*B*) up to 100 nM C3a, levels which are unlikely to be achieved in vivo, where C3a is rapidly inactivated by serum carboxypeptidase N (44). Hydrolyzed C3 (C3H_2_O) but not native C3 has been shown to be internalized from the extracellular space by endocytosis in diverse types of cells and appears to be cleaved by intracellular proteases, producing C3a fragments (45). We made a similar observation (46) and in addition to C3 internalization reported that extracellularly generated C3a via alternative pathway C3-convertase action can be transferred to the intracellular space, enter the nucleus, bind to DNA, and might regulate DNA transcription (46). Given that expression of cytosolic C3 fully reversed the increased IL-1β sensitivity of C3-knockouts, it is therefore possible that exogenously added C3/C3a might enter the intracellular environment and exert effects via intracellular C3/C3a detection mechanism. C3aR KD or deletion had no effect on survival of INS-1 cells, although we can not rule out an alternative receptor for intracellular C3 / C3a, or direct effects on gene expression via DNA interaction as reported previously (46).

Activation of c-Jun triggers proapoptotic signaling, and c-Jun inhibition has been proposed as a target for T2D (47). Our observation of cytosolic C3 countering activation of c-Jun therefore strongly supports a cytoprotective nature of cytosolic C3 in favorable β-cell health under normal and stress conditions. β-cells are enriched in expression of IL1R, making them exquisitely sensitive to the IL1R ligands, IL-1α and IL-1β. Low levels of IL-1β appear to be beneficial for β-cell function; Transient postprandial of IL-1β stimulate insulin secretion (40), and IL1R is required for β-cell proliferation (48), and limits islet β-cell de-differentiation (49), indicating important beneficial physiological functions of IL1R signaling. We found no evidence that loss of C3 inhibits the beneficial effect of short-term, low-level IL-1β on insulin secretion in vivo. Nevertheless, IL-1β becomes toxic for β-cells and triggers β-cell death, if chronically present in higher doses (1). Intracellular cytosolic C3 might therefore serve as a molecular suppressor preventing this shift from physiological to pathophysiological responses, being rapidly and strongly up-regulated by IL1R stimulation. The strong co-upregulation of CFB expression could potentially lead to increased alternative pathway activation and production of extracellular C3a and other complement activation products, the detection of which are increased in prediabetic islets (20). C3 upregulation may therefore have two competing outcomes, with intracellular C3 conferring protection, while increases in C3 and local CFB secretion may contribute to islet inflammation, with detrimental long-term outcomes. This aspect of islet-derived complement expression deserves further investigation. Our in vivo model can not provide direct proof of the intracellular function within β-cells, but rather only a cell-intrinsic function. However, pancreatic islets are highly vascularized, and β-cells are in direct and intimate contact with blood serum in order to sensitively monitor blood glucose levels and therefore are surrounded by high levels of serum C3. It seems unlikely that C3 secreted from β-cells will significantly contribute to this local extracellular pool of C3, but rather that intracellular C3 is responsible for the differences in the two mouse genotypes, which have equivalent serum C3 levels. Our findings highlight the significance of specifically investigating the distinct functions of different forms of C3 in various cell types, as this pleiotropic protein possesses numerous functions that can sometimes have counteracting effects. Further future work and the development of more advanced models will be required to specifically address the intracellular compared to extracellular roles of C3.

Our findings therefore demonstrate additional roles for intracellular, cytosolic C3 in maintenance of β-cell health and show that increased intracellular expression of C3 confers protection to β-cells exposed to IL-β through its interaction with FRK. β-cell-intrinsic cytosolic C3 therefore protects against pathological IL-1β responses, safeguarding β-cell function and health.

## Materials and Methods

### Human Islets and Expression Data.

Islets from consenting human donors were obtained from the Nordic Network for Islet Transplantation (Uppsala University, Sweden), under approval of the ethics committees at Uppsala and Lund. Total RNA sequencing data from isolated pancreatic islets from 57 human donors were made available via the ExoDiab Human Tissue Lab. The dataset was chosen to include only data from islets that had been in culture for up to 2 d before RNA extraction, as islets cultured for a longer time take on a proinflammatory signature (16). The online IGW Islet Gene View resource (25) was accessed at https://mae.crc.med.lu.se/IsletGeneView/, and the top correlating expressed genes for C3 were generated by using the Gene coexpression tool.

### Human Islet Treatments.

Around 1,500 human pancreatic islets were cultured for 24 h in Opti-MEM (Gibco # 31985-054) medium with or without recombinant human IL-1β (1 ng/ml, Mabtech # 3415-1H-6). After 24 h, supernatants and lysates, prepared with RIPA buffer (150 mM NaCl, 50 mM Tris-HCl, pH 7.5, 1% NP40, 0.5% deoxycholate, and 0.1% SDS with protease inhibitor cocktail; Roche), were harvested for C3 and CFB analysis.

## Experimental Animals.

C3-floxed dTomato reporter mice on the C57Bl/6 N background were generously supplied by Professor Claudia Kemper, NIH, and crossed with RIP-Cre mice on the same background, to produce β-cell-specific knockout mice. For intraperitoneal (IP) glucose tolerance tests (IPGTT), littermate male or female mice were used at 25 wk of age. Recombinant mouse IL-1β (ImmunoTools) or PBS vehicle control was injected at 1 ng/g IP at 20 min before commencing IPGTT, which involved IP injection of 2 g/kg glucose, followed by measurement of blood glucose levels in blood sampled from the vena saphena at regular intervals, and measured using a portable AccuCheck glucose meter (Roche). Mice were fasted for 4 h before commencing the IPGTT. For multiple low-dose streptozotocin (STZ) induction of diabetes, 27-wk-old littermate male mice were injected IP daily for 5 d with 50 mg/kg STZ (Sigma), and nonfasted blood glucose measurements were taken daily. Islets were isolated from C3 KO mice (Jackson Lab, #029661) or WT littermates by collagenase perfusion of pancreases directly after the mice were killed, followed by incubation at 37 °C, dispersal by mechanical shaking, and washing of islets in HBSS, followed by picking of islets from exocrine material under a stereo microscope. Insulin secretion assays were carried out using five islets per well of a 96-well plate, incubated 1 h in low glucose (2.8 mM) in secretion assay buffer (SAB) (40 mM HEPES, pH 7.2, 114 mM NaCl, 4.7 mM KCl, 1.2 mM KH_2_PO_4_, 1.16 mM MgSO_4_, 2.5 mM CaCl_2_, 25 mM NaHCO_3_, and 0.1% BSA), followed by incubation at 1 h at either low or high glucose (16.7 mM) SAB, before collection of supernatants and measuring insulin by ELISA (Mercodia). All animal experiments were approved by the Malmö-Lund ethical committee under permit number 20069/2020. Sources of rodent islet RNA from db/db and Akita mice, GK rats and Biobreeder rats, and their controls, were described in a previous study (16), and stored at −80 °C until the current study. RNA integrity was confirmed using an Agilent BioAnalyzer before use.

### Immunofluorescence in Paraffin-Embedded Tissue.

For tissue staining, 5-µm sections were deparaffinized in xylene twice for 10 min, 100% ethanol twice for 5 min, 95% for 5 min, and 70% for 5 min and then rehydrated in PBS three times for 3 min. Tissues were subjected to heat-induced antigen retrieval in 10 mM sodium citrate buffer (pH 6.0) for 10 min at 90 °C. Tissue sections were then permeabilized and blocked with 5% normal donkey serum in 5% Triton X-100 in PBS for 2 h at 4 °C. Tissues were then incubated with primary antibodies in blocking buffer, anti-Insulin/Proinsulin (PROGEN, cat. no. 16049) 1:200, anti-FRK (A gift from Professor Welsh, Uppsala University) 1:500, and rabbit anti-glucagon Nordic Biosite, #15954-1-AP) 1:500, overnight at 4 °C, washed three times in 0.2% Triton X-100 in PBS, and incubated with secondary antibodies (Thermo Fisher Scientific Inc.) for 4 h at 4 °C. Slides were then washed three times in PBS with 0.2% Triton X-100, and a coverslip mounted with mounting media containing DAPI (ThermoFisher) before imaging by confocal microscopy using a Zeiss LSM 510 Meta Confocal microscope with 20× magnification lens. Images were analyzed using Ilastik and ImageJ software.

### INS-1 cells culture.

INS-1 832/13 cells (a kind gift from C. Newgard, Duke University, Durham, NC, USA) (50) were cultured in RPMI1640 (HyClone # SH30255.01) supplemented with 10% fetal bovine serum (ATCC), 100 U/ml penicillin, 100 mg/mL streptomycin (HyClone), 2 mM L-glutamine (HyClone), 1 mM sodium pyruvate (HyClone), and 50 µM β-mercaptoethanol (Sigma-Aldrich). Cells were regularly tested for *Mycoplasma.* All experiments with IL-1β were performed in culture medium with 24 h of IL-1β exposure (1 ng/mL, Mabtech # 3415-1H-6).

### Generation of gene-edited INS-1 clones.

C3 knockout clones were produced as previously described (16). ΔATG1 clones were raised by cloning a guideRNA TTTACCATGGGACCCACGTCAGG, designed using CHOPCHOP (51), which targets the C3 gene directly downstream of the canonical ATG start site. This guideRNA was cloned into the pX459 plasmid (52), a gift from Dr Feng Zhang (Addgene plasmid # 62988; http://n2t.net/addgene:62988; RRID:Addgene_62988). After transfection, single clones were isolated by limiting dilution, screened for loss of C3 secretion by ELISA of supernatants, and the successful indel verified by amplifying and sequencing the locus using forward primer TTGTGAACAGCTTAGGAAACCA and reverse primer ATAAGAAACTGCACCCCAAGAA. INS-1 cells stably re-expressing human C3 were generated by transfection of C3-knockout clones with human C3 cDNA cloned into pSELECT.

### INS-1 Treatments: RNA Interference.

INS-1 cells were plated out at a density of 1 × 10^5^ cells/well in a 24-well plate. The following day, cells were transfected with a final concentration of 50 nM of C3 ON-TARGET plus siRNA and corresponding negative control siRNA (Dharmacon), using lipofectamine RNAiMAX, according to protocol (Thermo Fisher Scientific). After 24 h of incubation, cells were treated or not with IL-1β (1 ng/mL, Mabtech) for another 24 h.

### C3/C3a Treatment.

Cells seeded at 1.5 × 10^5^ cells/well in a 24-well plate were coincubated with IL-1β and sources of C3 at the following concentrations: normal rat serum (NRS) or heat-inactivated (HI)-NRS (10%), purified C3 (50 µg/mL, CompTech) and C3a (10, 50 and 100 nM, R&D system) for 24 h. Control cells not treated with siRNA or C3/C3a were seeded at 1.5 × 10^5^ cells/well in a 24-well plate, and the following day, IL-1β (1 ng/mL, Mabtech) was added for 24 h. STZ treatment of INS-1 cells was carried out at given concentrations for 24 h. Cells were then harvested by incubation with trypsin (HyClone), before viability assessment by Annexin-V-APC (ImmunoTools) binding by flow cytometry. For the autophagy study, after IL-1β stimulation for 24 h, cells were treated with 50 nM of chloroquine (Sigma) for 3 h before harvesting.

### Proximity Ligation Assays.

INS-1 cells were plated on a 8-well slide with removable chambers (IBIDI 80841), 0.5 × 10^5^ cells per well in 300 µL of culture medium. The slides were incubated at 37 °C for 48 h. Cells were transfected with human FRK cDNA cloned into pcDNA3, using lipofectamine 3,000:0.6 µL of lipofectamine, 0.6 µL of P3000 reagent, and 0.3 µg DNA per well following the manufacturer’s instructions. The following day, cells were processed for PLA staining using Duolink Proximity Ligation Assays (Merck) according to kit instructions. Primary antibodies were added at 2 µg/mL in blocking buffer: mouse anti-human C3/C3a (Hycult HM2073) and goat anti-FRK (R&D AF3766). Slides were incubated with primary antibodies overnight at 4 °C in a humidity chamber. After following the PLA procedure, slides were mounted with a coverslip using Duolink In Situ mounting medium with DAPI. For PLA imaging of primary mouse islets, freshly isolated islets were fixed with 4% PFA, permeabilized with 0.2% Triton X-100 in PBS for 10 min, then PLA carried out using the tissue Naveniflex kit (Navinci), according to kit instructions, using rabbit anti-mouse FRK and goat anti-mouse C3 (ICN #55500) primary antibodies. Z-stack images were taken using a Zeiss LSM 510 Meta confocal microscope, with DAPI and Cy3/Atto647 channels on a 63X magnification. Multiple images of each condition were taken. Using the Zeiss Zen software, Maximum Orthogonal Projection was performed to combine all the slices of the z-stack. PLA signals were counted on the Cy3/Atto647 channel of projections using the automated particle analysis function of ImageJ. DAPI was used to count the number of cells in each image.

### Cell Viability Tests.

For flow cytometry, cells were incubated in 50 μL of buffer (10 mM HEPES, 150 mM NaCl, 5 mM KCl, 1 mM MgCl_2_, 2 mM CaCl_2_, 0.02% sodium azide, and 0.5 % BSA, pH 7.4) with Annexin V-APC (1:50, ImmunoTools #31490016) and Viaprobe (1:20, BD Bioscience #555815) for 15 min on ice. After dilution up to 250 mL, samples were run on CytoFlex (Beckman Coulter), and data were analyzed with CytExpert Version 2.0. Alamar Blue (Thermo Fisher Scientific) viability reagent (50 μL) was added directly to cells, after incubation with IL-1β for 24 h. After incubation for 3 h, fluorescence intensity was measured using a fluorescence excitation wavelength of 570 nm and an emission of 590 nm.

### Quantitative PCR.

RNA was extracted using the RNeasy Plus Mini kit (Qiagen), and cDNA was synthesized using oligo-dT primers and SuperScript IV (Invitrogen). Quantitative PCR was performed with specific TaqMan probes (Applied Biosystem) and Viia7 Real-Time PCR system (Thermo Fisher). Expression levels of C3, CFB, CFD, and caspase-3 in INS-1 cells and islets were calculated after normalization with the geometric mean of the housekeeping gene hypoxanthine-guanine phosphoribosyltransferase (HPRT). Data are presented as a relative expression (ΔCt) or fold change expression (ΔΔCt). CFB and CFD in mice and rats were normalized to Cyclophilin A. Rodent islet RNA was obtained from rodent diabetic models as described previously (16).

### Rat C3 ELISA.

INS-1 cells were plated out at density of 1.5 × 10^5^ cells/well in a 24-well plate. The next day, IL-1β was added at a concentration of 1 ng/mL (Mabtech) for 24 h in culture medium. Supernatant and lysates, prepared using 30 μL of RIPA/well, were then harvested for ELISA: The microtiter plate was coated with anti-rat C3 (Hycult Biotech HP8022) diluted 1:50 in 75 mM sodium carbonate, pH 9.6, at 4 °C overnight. After 1-h blocking in quench buffer [3% fish gelatin in immunowash (50 mM Tris-HCl, 150 mM NaCl, 0.1% Tween 20, pH 8.0)], a standard of normal rat serum (NRS, Abcam) and samples were applied (150 μL of supernatant and 20 μL of lysate) and incubated ON at 4 °C. Fractionated samples (isolated using the Mem-Per Plus eukaryotic protein extraction kit (Thermo Scientific), according to instruction) were loaded at an amount of 100 μL/well. C3 was detected with anti-C3, HRP conjugated (1:2,000, MP Biomedicals # 0855237). Bound enzyme was visualized with OPD (EM-EN-TEC). The plate was washed 4 × with immunowash between steps.

### Western Blots.

Supernatants or lysates prepared using RIPA containing protease inhibitor cocktail (Roche) were resolved by SDS-PAGE under reducing condition and transferred to PVDF membrane with the Trans-Blot Turbo system (Bio-Rad). Membranes blocked with quench buffer were probed with the following primary antibodies: anti-FB (1:5,000, CompTech #A235), anti-LC3 (1:2,000 Sigma #L7543), anti-cleaved caspase-3 (1:2,000, Cell Signaling Technology #9664), and anti-β-actin (1:1,000, Abcam #ab8226). Alternatively, membranes were blocked with 5% milk in immunowash and probed with anti-human C3 (1:5,000, CompTech #A213), anti-PTEN (1:1,000, Cell Signaling Technology #9188), anti-AKT (1:1,000, Cell Signaling Technology #4691), or anti-phosphoSerine AKT (1:2,000, Cell Signaling Technology #4060). Primary antibody incubation was followed by incubation with appropriate secondary antibody (Dako) conjugated with HRP and development with ECL reagent (Santa Cruz Biotechnology).

### Statistical Analysis.

All statistical analyses were performed using GraphPad Prism 8. Values are expressed as mean ± SD, with circles indicating individual repeats and average from two individual clones, unless indicated otherwise. Statistical tests applied are indicated in the figure legend. In all figures, *P < 0.05, **P < 0.01, ***P < 0.001, and **** *P* < .0.0001.

## Supplementary Material

Appendix 01 (PDF)

## Data Availability

All study data are included in the article and/or *SI Appendix*.
