# Supplementary material for: Intracellular C3 protects β-cells from IL-1β-driven cytotoxicity via interaction with Fyn-related kinase
Source: Proc Natl Acad Sci U S A. 2024 Feb 12;121(8):e2312621121. doi: 10.1073/pnas.2312621121 (PMC10895342; doi:10.1073/pnas.2312621121)
Supplement: Supplementary file 1 — Appendix 01 (PDF) [file pnas.2312621121.sapp.pdf]

PNAS

Supporting Information for:

Intracellular C3 protects β-cells from IL-1β-driven cytotoxicity via interaction with Fyn-related kinase (FRK).

Klaudia Kulak[1], Katarzyna Kuska[1], Lucie Colineau[1], Marina Mckay[1], Karolina Maziarz[1], Julia Slaby[1], Anna M Blom[1,2], Ben C King[1,2]

[1]Section of Medical Protein Chemistry, Department of Translational Medicine, Lund University, Malmö, 214-28 Sweden
[2]These authors contributed equally

Corresponding author: Anna Blom, Dept. of Translational Medicine, Lund University, anna.blom@med.lu.se

This PDF file includes:

Figures S1 to S4
Table S1

Fig. S1. Correlation of gene expression and donor characteristics in human islets:

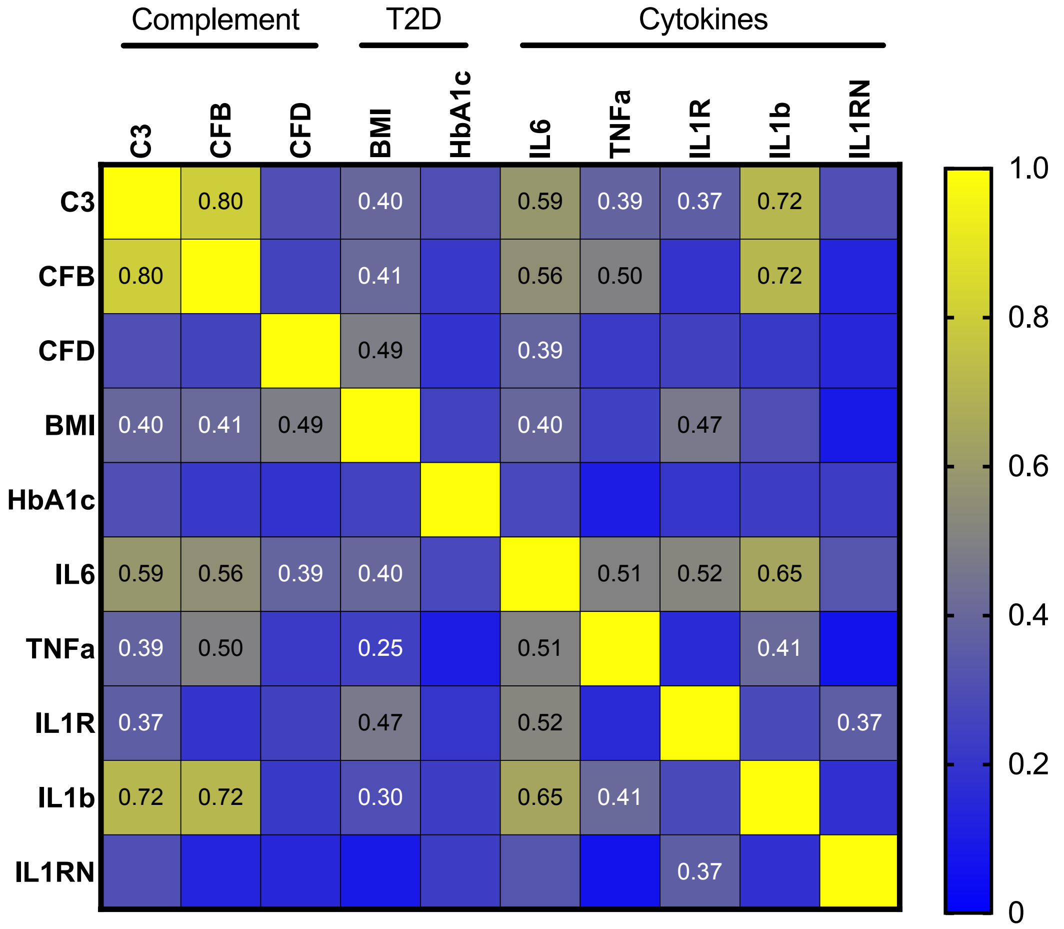

Correlation of expression levels of alternative pathway complement genes, donor T2D values (BMI and HbA1c), and cytokine gene expression levels, from RNA-Seq expression data from 58 human islet preparations, selected from a larger dataset by limiting to “fresh” islets that had only been in culture for up to 2 days before RNA extraction. This is due to the reported increase in pro-inflammatory gene signature of islets with increased time in culture. Spearman r correlation values are shown only for those correlations that had a statistical significance of $p < 0.005$.

Fig. S2. Sequencing results verifying ΔATG1 INS-1 clones.

A

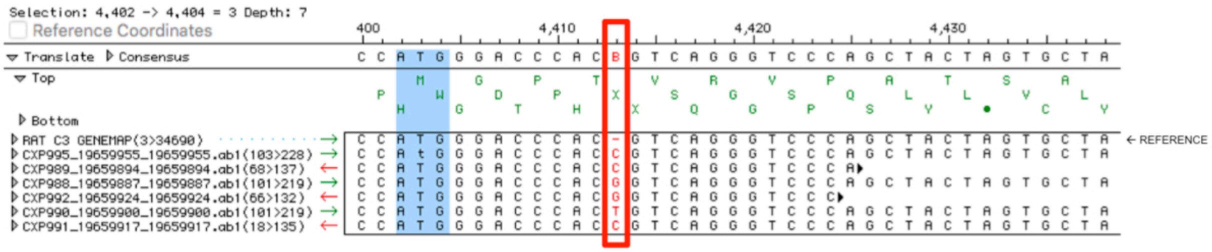

B Ref: MGPTSGSQLLVLLLLLASSLLALGSPMYSIITPNVLRLES...

ΔATG1: MGPTVRVPATSATAAVGQLPASSGEPHVLHHYSQCPAAGE*

A) Sequencing results from forward and reverse sequencing of the ATG1 locus from three individual clones. Compared to the reference Rat C3 sequence (top row), and as a result of non-homologous end joining repair, all three clones have single nucleotide insertions at the predicted double-stranded break site, 3-4 nucleotides upstream of the protospacer adjacent motif (PAM) *Streptococcus pyogenes* Cas9 cleavage site (AGG). The ATG1 start site is highlighted, and the identified indel site is within the red box. B) Comparison of the rat C3 reference amino acid sequence (top row), with the peptide sequence from sequenced ΔATG1 clones (below). An early frameshift caused by the single nucleotide insertion leads to an altered peptide sequence and early stop codon, indicated by an asterisk. An alternative translational start site downstream of the signal peptide leads to production of the cytosolic C3 isoform lacking the signal peptide, as detected by ELISA (Figure 4) (see also Kremlitzka et al,(1)).

Fig. S3. IL-1β treatment does not induce conventional autophagy

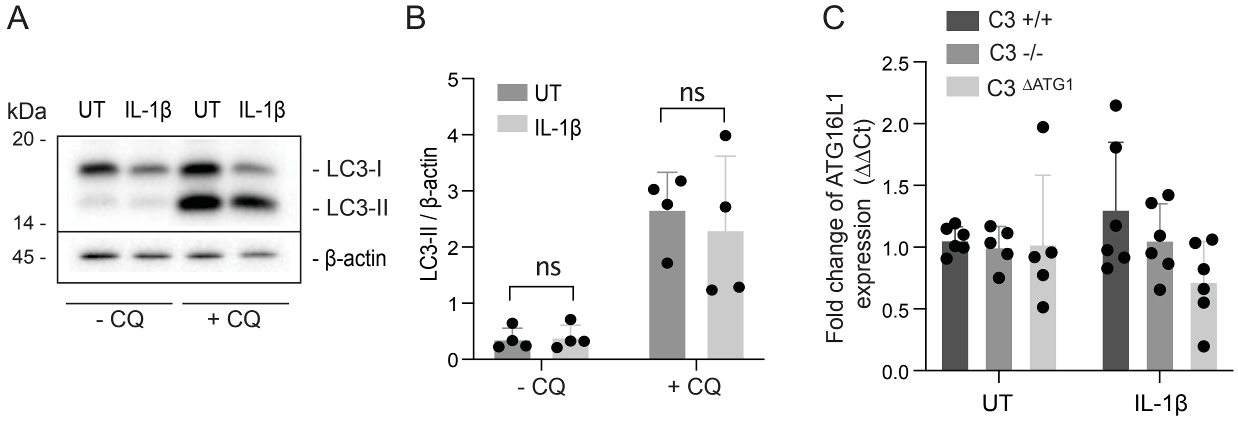

A) Representative blot for LC3-I/II in INS-1 cells, with or without IL-1β stimulation and chloroquine (CQ) incubation. B) Quantification of LC3-II density normalized to β-actin, showing that autophagy turnover is not accelerated in presence of IL-1β, indicated by a lack of LC3-II accumulation comparing to cells untreated with IL-1β. C) qPCR of ATG16L1 expression level in INS-1 clones +/- IL-1β treatment. B, C) 2-way ANOVA, Bars display mean +/- SD with circles representative of 4 individual repeats (B) and in C) circles indicate 3 repeats of 2 individual INS-1 clones.

Fig. S4. Proximity Ligation Assay between human C3 and FRK in INS-1 cells:

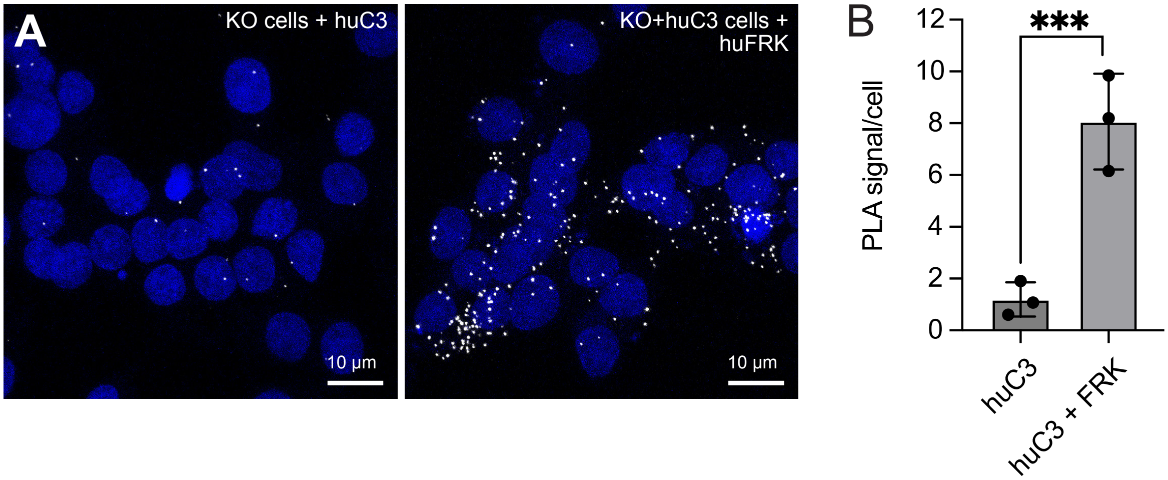

A) PLA results from INS-1 cells stably expressing both human FRK and human C3, and transfected with human FRK, with non-transfected cells as negative controls, demonstrating colocalization. B) Quantification of PLA results from 3 independent repeats. Blue: DAPI staining of nuclei, white: colocalization puncta between C3 and FRK.

Table S1. Top 100 genes correlating with human C3 expression in human islets. From Islet GeneView co-expression tool, https://mae.crc.med.lu.se/IsletGeneView (2).

Target	Correlation	Symbol
**ENSG00000243649**	**0.829679623**	**CFB**
ENSG00000149131	0.784102581	SERPING1
ENSG00000112096	0.783985386	SOD2
ENSG00000100665	0.781986404	SERPINA4
ENSG00000124145	0.780599117	SDC4
ENSG00000053918	0.780471914	KCNQ1
ENSG00000197249	0.77632735	SERPINA1
ENSG00000273259	0.774726875	SERPINA3
ENSG00000139178	0.772985903	C1RL
ENSG00000062038	0.767868882	CDH3
ENSG00000086062	0.76040908	B4GALT1
ENSG00000172016	0.745297037	REG3A
ENSG00000196136	0.743376345	SERPINA3
ENSG00000132693	0.739304342	CRP
ENSG00000205403	0.733031217	CFI
ENSG00000104140	0.726095037	RHOV
ENSG00000140279	0.722072975	DUOX2
ENSG00000182054	0.722016161	IDH2
ENSG00000155629	0.721274941	PIK3AP1
ENSG00000115009	0.719914612	CCL20
ENSG00000213886	0.719733685	UBD
ENSG00000102837	0.715617969	OLFM4
ENSG00000136872	0.71398353	ALDOB
ENSG00000076716	0.710254946	GPC4
ENSG00000166825	0.708267508	ANPEP
ENSG00000132703	0.707029278	APCS
ENSG00000071575	0.705294069	TRIB2
ENSG00000128849	0.70252643	CGNL1
ENSG00000188488	0.699115867	SERPINA5
ENSG00000152229	0.694579784	PSTPIP2
ENSG00000065485	0.693476394	PDIA5
ENSG00000125538	0.693298109	IL1B
ENSG00000135052	0.692297308	GOLM1
ENSG00000128487	0.68974264	SPECC1
ENSG00000135048	0.687590273	TMEM2

ENSG00000077274	0.687565554	CAPN6
ENSG00000169403	0.687468084	PTAFR
ENSG00000144908	0.686453565	ALDH1L1
ENSG00000138798	0.686381679	EGF
ENSG00000130066	0.685289951	SAT1
ENSG00000124875	0.684669777	CXCL6
ENSG00000115008	0.68325282	IL1A
ENSG00000123243	0.677331442	ITIH5
ENSG00000198624	0.676507439	CCDC69
ENSG00000148702	0.675984237	HABP2
ENSG00000134247	0.675917987	PTGFRN
ENSG00000101825	0.675090279	MXRA5
ENSG00000176826	0.675004267	FKBP9P1
ENSG00000137462	0.673643612	TLR2
ENSG00000047457	0.672327781	CP
ENSG00000103534	0.671443581	TMC5
ENSG00000273604	0.670906184	C17orf96
ENSG00000171557	0.669251069	FGG
ENSG00000171560	0.669196587	FGA
ENSG00000055955	0.668183731	ITIH4
ENSG00000163485	0.667268233	ADORA1
ENSG00000184012	0.666992352	TMPRSS2
ENSG00000007062	0.664722795	PROM1
ENSG00000134873	0.663698187	CLDN10
ENSG00000162366	0.662638651	PDZK1IP1
ENSG00000178826	0.661630715	TMEM139
ENSG00000168961	0.661397169	LGALS9
ENSG00000163435	0.659679812	ELF3
ENSG00000118707	0.658579556	TGIF2
ENSG00000008517	0.658007743	IL32
ENSG00000070404	0.655586754	FSTL3
ENSG00000171236	0.651207574	LRG1
ENSG00000123838	0.650982239	C4BPA
ENSG00000169991	0.65001797	IFFO2
ENSG00000118322	0.649693426	ATP10B
ENSG00000143061	0.649511873	IGSF3
ENSG00000162817	0.649469906	C1orf115
ENSG00000092621	0.647709998	PHGDH
ENSG00000169752	0.646672386	NRG4

ENSG00000235142	0.644700086	RP1-60O19.1
ENSG00000188257	0.644098876	PLA2G2A
ENSG00000150551	0.643091828	LYPD1
ENSG00000171940	0.642332075	ZNF217
ENSG00000165929	0.641668303	TC2N
ENSG00000095585	0.641438274	BLNK
ENSG00000088280	0.641169023	ASAP3
ENSG00000162738	0.641157207	VANGL2
ENSG00000133048	0.640272636	CHI3L1
ENSG00000121858	0.639636089	TNFSF10
ENSG00000163631	0.638002224	ALB
ENSG00000023445	0.63579396	BIRC3
ENSG00000240583	0.635471139	AQP1
ENSG00000080293	0.635212464	SCTR
ENSG00000166920	0.634698452	C15orf48
ENSG00000070731	0.634415739	ST6GALNAC2
ENSG00000132698	0.634315083	RAB25
ENSG00000066468	0.634147421	FGFR2
ENSG00000188313	0.633974242	PLSCR1
ENSG00000224389	0.633483654	C4B
ENSG00000164976	0.632471362	KIAA1161
ENSG00000163900	0.631958877	TMEM41A
ENSG00000140274	0.631791629	DUOXA2
ENSG00000142949	0.629488443	PTPRF
ENSG00000189067	0.629451369	LITAF
ENSG00000184454	0.629111417	NCMAP

SI References:

1. M. Kremlitzka *et al.*, Alternative translation and retrotranslocation of cytosolic C3 that detects cytoinvasive bacteria. *Cell Mol Life Sci* **79**, 291 (2022).
2. O. Asplund *et al.*, Islet Gene View-a tool to facilitate islet research. *Life Sci Alliance* **5** (2022).
